# High throughput microscopy identifies bisphenol AP, a bisphenol A analog, as a novel AR down-regulator

**DOI:** 10.18632/oncotarget.7655

**Published:** 2016-02-24

**Authors:** Fabio Stossi, Radhika D. Dandekar, Michael J. Bolt, Justin Y. Newberg, Maureen G. Mancini, Akash K. Kaushik, Vasanta Putluri, Arun Sreekumar, Michael A. Mancini

**Affiliations:** ^1^ Department of Molecular and Cellular Biology, Baylor College of Medicine, Houston, TX 77030, USA

**Keywords:** androgen receptor, bisphenol A analogs, high throughput microscopy, AR-V7, castrate resistant prostate cancer

## Abstract

Prostate cancer remains a deadly disease especially when patients become resistant to drugs that target the Androgen Receptor (AR) ligand binding domain. At this stage, patients develop recurring castrate-resistant prostate cancers (CRPCs). Interestingly, CRPC tumors maintain dependency on AR for growth; moreover, in CRPCs, constitutively active AR splice variants (*e.g.,* AR-V7) begin to be expressed at higher levels. These splice variants lack the ligand binding domain and are rendered insensitive to current endocrine therapies. Thus, it is of paramount importance to understand what regulates the expression of AR and its splice variants to identify new therapeutic strategies in CRPCs. Here, we used high throughput microscopy and quantitative image analysis to evaluate effects of selected endocrine disruptors on AR levels in multiple breast and prostate cancer cell lines. Bisphenol AP (BPAP), which is used in chemical and medical industries, was identified as a down-regulator of both full length AR and the AR-V7 splice variant. We validated its activity by performing time-course, dose-response, Western blot and qPCR analyses. BPAP also reduced the percent of cells in S phase, which was accompanied by a ~60% loss in cell numbers and colony formation in anchorage-independent growth assays. Moreover, it affected mitochondria size and cell metabolism. In conclusion, our high content analysis-based screening platform was used to classify the effect of compounds on endogenous ARs, and identified BPAP as being capable of causing AR (both full-length and variants) down-regulation, cell cycle arrest and metabolic alterations in CRPC cell lines.

## INTRODUCTION

Androgen receptor (AR) is a member of the nuclear receptor (NR) superfamily regulating the expression of genes that are important in development, differentiation, and cancer progression, especially of the prostate [1]. Upon binding to its endogenous hormones (*i.e.,* dihydrotestosterone (DHT)), AR undergoes a conformational change, sheds heat shock proteins, dimerizes, and translocates from the cytoplasm to the nucleus where it binds specific DNA sequences, thus modulating gene transcription [2, 3]. Prostate cancers are generally treated by blocking AR activity using some form of androgen ablation therapy that targets the AR ligand binding domain (LBD); this approach has been shown to be therapeutically very effective, at least initially [1, 4]. However, many patients become resistant, with the disease transitioning into castrate-resistant prostate cancers (CRPCs) for which there are still no effective treatments [5]. Interestingly, CRPC tumors do not lose dependency on the AR for growth. Another layer of complexity in CRPCs is caused by the appearance of constitutively active AR splice variants, which lack the C-terminal LBD, and therefore, cannot be targeted by current therapies [6–8]. For example, the AR-V7 variant has been linked to poor prognosis in CRPC patients [9]. Identifying small molecules that reduce AR levels is an attractive avenue for treatment of CRPC patients, especially since all available therapies leave untouched any of the splice variants.

We have previously developed and utilized high throughput microscopy-based platforms, high content analysis (HCA) and high content screening (HCS) to define and quantify many mechanistic steps of NR activities [2, 3, 10–12]. More recently we used these approaches to characterize the endocrine disruptor (EDC) Bisphenol A and related analogs (BPXs) for their effects upon multiple mechanistic steps involved in Estrogen Receptor (ER)α or ERβ functions [13, 14]. Here, we expanded these efforts to include analysis of several breast and prostate cancer cells specifically looking for the effects of EDCs on AR levels and/or localization. We chose to examine a panel of EDCs in an AR context as there are examples of successful new drug candidates that derive from this group of compounds. Most notably, EPI-001 (and its derivatives) was identified from screening a marine sponge library and its unconventional mechanism of action in prostate cancer is still under intensive investigation [15–17]. In this study, primary screening led to the identification of bisphenol AP (BPAP), a BPA analog already present in the environment and extensively used as a plasticizer, as a novel regulator of AR. We show that BPAP exhibited down-regulatory effects upon full length AR (f.l.AR) and AR-V7 splice variant in commonly used CRPC model cell lines, with little effect on AR levels in cell lines that do not express the AR variants (*i.e.*, LNCaP, MCF-7). BPAP also caused cell cycle arrest, metabolic changes and reduction in colony formation thus possessing the potential as a CRPC growth-inhibiting compound, albeit with low potency. In conclusion, our high throughput microscopy based platform was used to identify BPAP as a potential lead compound that influences: a) AR levels and activity (including splice variants) in CRPC models, b) cell cycle progression/arrest and anchorage-independent growth and, c) cell metabolism.

## RESULTS

### Characterization of endogenous AR protein levels and localization in a panel of breast and prostate cancer cell lines by high throughput microscopy

HTM/HCA coupled with single cell image analysis is a fast and powerful way to analyze effects of a compound on a target protein [2, 14]. In this study, we were interested in determining if we could use this method to classify known ligands of AR, to potentially identify novel modulators of AR levels/translocation, and also to determine cell type specific effects without the need of engineered cell lines expressing epitope-tagged AR.

**Figure 1:**
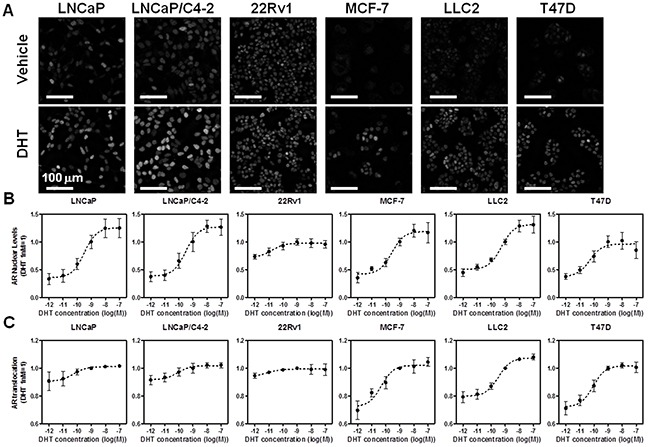
High throughput microscopy-based analysis of endogenous AR nuclear level and translocation across prostate and breast cancer cell lines **A.** Representative images of the cell lines used in HTM labeled with AR441 antibody after 24 hrs of treatment with either vehicle (top) or 1 nM DHT (bottom). **B–C.** DHT six point dose response and analysis of AR nuclear levels (B), or translocation (C), as determined by HTM and image analysis protocols

With these goals in mind we selected a panel of prostate and breast cancer cell lines and used automated image analysis routines to quantify endogenous AR nuclear levels and localization in response to DHT (Figure 1A–1C), at the single cell level, using a validated N-terminal AR antibody (AR441). Similar results were also obtained using a different antibody (Supplementary Figure S1). Dual immunofluorescence demonstrated excellent correlation between the two antibodies at the single cell level (Spearman's r>0.7, Supplementary Figure S1C, 22Rv1 shown as an example). The prostate cancer cell lines that were chosen represent well-characterized tumor states (*i.e.,* androgen dependent (LNCaP), androgen sensitive (LNCaP/C4-2) and castration resistant (22Rv1)), and also include examination of mutated forms of AR (*i.e.,* LNCaP) and AR splice variants (*i.e.,* 22Rv1). As a comparison for the prostate cancer cell lines, we selected luminal A breast cancer cell lines (MCF-7, MCF-7 resistant to Tamoxifen (LLC2), and T47D). Through well-established image analysis routines (as described in *Materials and Methods,* and [2, 3, 10, 18]]), we measured AR nuclear levels (which results from the combination of nuclear translocation and protein stability) and nuclear translocation (the ratio between nuclear and cytoplasmic signals) comparing vehicle and DHT treatment for 24 hrs (Figure 1A and Supplementary Figure S1 show example images of AR level and localization across all cell lines after dual immunofluorescence using AR441 and AR N-20 antibodies). We performed a six point dose response for DHT treatment (10pM to 100nM) to gain more information on the sensitivity of DHT action on AR nuclear levels and translocation across the cell lines (Figure 1B–1C). The data indicate that, as expected, AR expression and translocation increased following treatment with DHT to different degrees in all the cell lines except 22Rv1. This is largely due to the high expression of several constitutively nuclear AR splice variants (*e.g.,* AR-V7) lacking the LBD that are recognized by the N-terminal AR antibody. Cytoplasmic/nuclear translocation was more evident in the breast cancer cell lines, suggesting that these cells may be more useful for screening compounds that affect AR cytoplasmic/nuclear ratio, and further support testing of multiple model systems to gain a clearer picture of the effects of different classes of compounds.

### Cell type specific effects of AR antagonists determined by HTM

**Figure 2:**
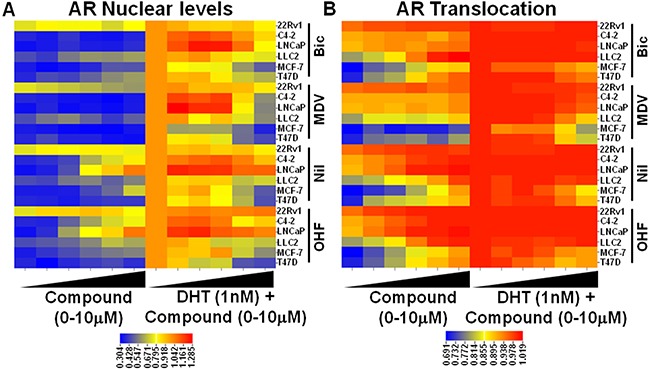
Characterization of established AR antagonists by HTM across multiple cell lines Cells were treated with a six point dose response of known AR antagonists +/− 1nM DHT and AR nuclear levels **A.** and translocation **B.** were measured by HTM and represented as a heatmap.

We treated the six cell lines with a panel of known AR antagonists that have been approved for patient treatment: bicalutamide (Bic), nilutamide (Nil), MDV3100 (enzalutamide, MDV), and hydroxyflutamide (OHF) (Figure 2). Each drug was used in a six point-dose response for 24 hrs either alone (Compound), or in combination with 1 nM DHT (DHT+Compound). Figure 2A–2B show heatmaps representing the dose response for each compound alone or in combination with DHT, and their effect upon AR nuclear levels (Figure 2A) and translocation (Figure 2B).

From viewing the heatmap in Figure 2, it is evident that, when used alone, some compounds elicit DHT-like activity in terms of AR protein stabilization (*e.g.,* nilutamide, OHF) and translocation, especially in prostate cancer cells (LNCaP and C4-2) as compared to breast cancer lines. This was also true when the antagonists were combined with DHT (Figure 2). MDV3100 (enzalutamide) was the only compound that blocked the DHT effect upon AR stabilization in all the cell lines (except 22Rv1), albeit at different levels. In terms of changes in nuclear localization, most of these drugs caused nuclear trafficking of AR [3], which was expected since they bind to the AR LBD and their mechanism of inhibition of AR action occurs largely at the transcriptional output level. MDV3100 was also an exception to this observation, as it reduced DHT-induced nuclear localization, especially in MCF-7 and T47D breast cancer cells, and less so in the prostate cancer cell lines. Thus, combination of imaging-based readouts across multiple cell lines is capable of discriminating between different drugs, both in “agonistic” and “antagonistic” treatment regimes; further, the high throughput nature of the assay makes it amenable to larger screening efforts for ligand classification and structure activity relationship (SAR) studies.

### Analyzing environmental compounds by AR HTM

**Figure 3:**
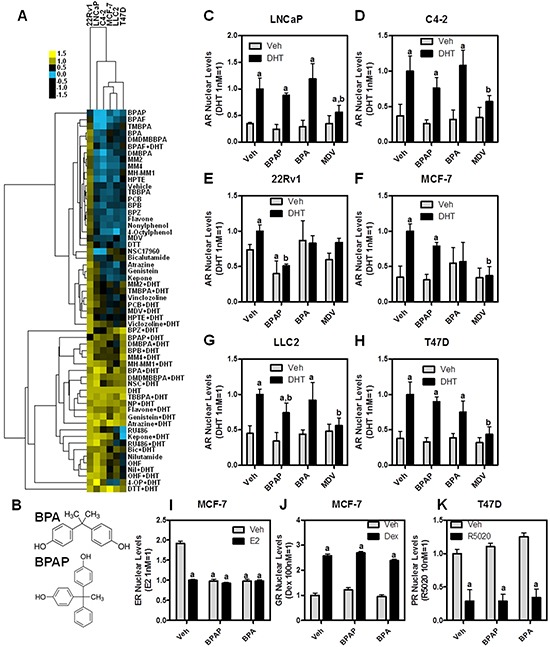
High throughput screening (HTS) of endocrine disruptors identifies BPAP as an AR down-regulator in 22Rv1 cells **A.** Cluster dendrogram of the HTS performed in six cell lines treated for 24 hrs with the indicated compounds at 10μM, and then immunolabeled with an AR antibody. Heatmap represents AR nuclear levels (yellow = high, blue = low); **B.** chemical structure of BPA and BPAP; **C–H.** raw data from panel A) showing a direct comparison between BPA, BPAP and MDV3100; I-K) BPAP or BPA treatment (10μM for 24h) +/− hormonal stimuli (E2, dexamethasone or R5020) followed by immunolabeling for ERα I. GR J. or PR K. *a*: p<0.05 *vs.* vehicle; *b*: p<0.05 *vs.* DHT.

As part of a larger series of studies examining the activity of EDCs on NR activities [14], we tested a subset of these compounds, some of which have been shown to affect AR activity (*i.e.*, vinclozoline), together with a set of BPA analogs using the platform we described above. BPA and some of its derivatives have been previously shown to affect AR action [19–22], mostly as antagonists, prompting the hypothesis that functional differences between EDCs could be distinguished using our microscopy-based approach and/or identify new or cell type-specific effects of these compounds. This idea was tested by treating our cell line panel with the indicated compounds +/− DHT for 24 hrs, and then measuring AR nuclear levels as an end point. All the data on changes in AR nuclear levels were clustered using Euclidean distance and represented as a dendrogram (Figure 3A). As expected, the clustering revealed that 22Rv1 cells, which express relatively high levels of nuclear-resident AR splice variants, constituted a separate branch, with all the others cell lines being less distinct. In terms of the ligand tree cluster, it is apparent how two large groups were defined: one centered on the vehicle control and the other on DHT treatment, thus showing that our clustering method efficiently separated vehicle vs. agonist treated samples. There were two main subgroups in the vehicle branch; the first subgroup centered on the vehicle control and was comprised of compounds that showed little effect on AR levels when treated alone. A notable exception was Bisphenol AF (BPAF), a BPX that partitions to this region of the dendrogram when used alone or in combination with DHT, thus indicating it is a potentially good AR antagonist across all cell lines (Figure 3A). Indeed, BPAF has been reported to bind to AR and to reduce testosterone levels in rats [19, 22]. The second subgroup contained both compounds with low activity when treated alone and with known AR antagonists (*i.e.*, MDV3100) used in combination with DHT.

The DHT treatment-containing group was primarily comprised of compounds that do not have much impact on antagonizing DHT across cell lines; however, there are few interesting observations. First, when used individually, nilutamide, OHF, and RU486 were present in this cluster indicating they cause similar increases in AR nuclear levels as DHT. RU486 was particularly interesting because its effect upon nuclear AR levels was cell type specific, with no effect in T47D and maximal effect in LNCaP. Two other cell type-specific compounds were identified:1) bisphenol Z (BPZ), a BPX that was antagonistic only in LNCaP and C4-2 prostate cell lines; and, 2) 4-octylphenol (4-OP), which had antagonistic activity only in MCF-7 and LLC2 breast cancer cell lines.

Collectively, our experimental interrogation of compound effects upon AR nuclear levels is thus capable of classifying ligands, and is helpful in identifying cell type-specific responses.

### BPAP induces down-regulation of full-length AR and truncated splice variants in CRPC cells

From the cluster analysis in Figure 3A, Bisphenol AP (BPAP) was readily identified as an outlier since it was the only compound tested that reduced AR nuclear levels in 22Rv1 cells, both in the presence or absence of DHT. The structures of BPAP and BPA are shown in Figure 3B. In Figure 3C–3H, BPAP was directly compared to MDV3100 and BPA in terms of modulating AR nuclear levels across cell lines. Our data indicates that BPAP has cell-specific effects, showing basal down-regulation of nuclear AR only in 22Rv1 cells; further, BPAP also statistically-reduced the DHT effect in 22Rv1 and MCF-7/LLC2. In comparison, MDV3100 and BPA treatments showed more widespread (MDV) or minimal (BPA) effects across the cell lines.

Because many EDCs are capable of affecting other nuclear receptors, we tested BPAP effects upon modulating the levels of several endogenous NRs (ER, glucocorticoid receptor, GR, and progesterone receptor, PR) in breast cancer cells (Figure 3I–3K). As expected, and as we have shown previously [14], BPAP is an ER binding compound causing reduction of nuclear ER after 24hrs, similar to E2 treatment (Figure 3I). No effect was observed on GR or PR nuclear levels, either when cells were treated with BPAP alone or in combination with their respective agonists (Figure 3J–3K).

**Figure 4:**
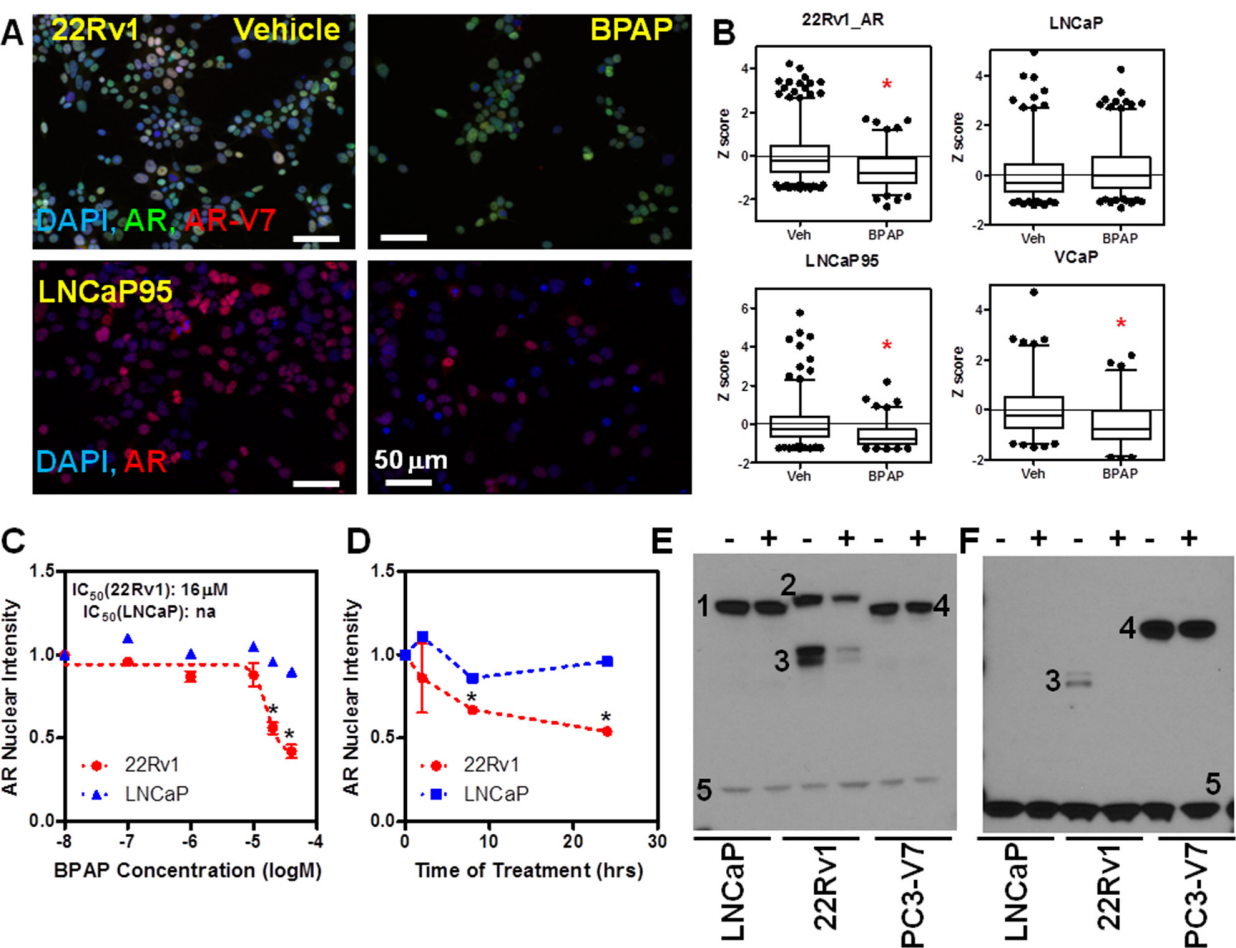
BPAP reduces AR full length and AR-V7 protein level in CRPC cell models **A.** Representative immunofluorescence images showing total AR and AR-V7 nuclear levels in 22Rv1 (A, top) and total AR in LNCaP95 (A, bottom) cells treated with vehicle or 20μM BPAP for 24 hrs. **B.** Single cell analysis of AR nuclear levels in 22Rv1, LNCaP95, VCaP and LNCaP (>1000 cells/condition) represented as box plot. *p<0.01 vs. vehicle. **C–D.** BPAP dose response and time course analysis in 22Rv1 cells by AR IF. **E–F.** Western blot in LNCaP, 22Rv1 and GFP-AR-V7:PC3 using AR (E) and AR-V-7 (F) antibodies showing reduction of AR (full length and variants) only in 22Rv1. Cells were treated with 20mM BPAP for 24 hrs. In the Western blots, 1 indicates full length AR, 2 full length AR with DBD duplication that is expressed in 22Rv1, 3 AR variants (AR-V7 in F), 4 GFP-AR-V7, 5 GAPDH.

Because the total AR protein in 22Rv1 is comprised of both a mutated full-length AR and constitutively active AR splice variants, we sought to determine if BPAP was capable of reducing all AR species across AR variant expressing cell lines [23]. For this reason we tested AR variants-containing cell lines (22Rv1, LNCaP95 and VCaP) by immunofluorescence using the AR441 antibody, and we also immunolabeled 22Rv1 cells with an AR-V7 specific antibody (Figure 4A–4B). To validate the specificity of the AR-V7 antibody, we engineered the AR negative PC-3 cell line to express GFP-AR-V7 and verified that the AR-V7 antibody was specific and amenable to imaging (Supplementary Figure S2A). Representative images of 22Rv1 and LNCaP95 cells are shown in Figure 4A. Single cell image analysis [3, 14, 18] revealed that a 24 hr treatment of BPAP resulted in a significant decrease of AR protein in all three AR variant-containing cell lines, but not LNCaP, which we used as control (Figure 4B).

We then performed a six-point dose-response (Figure 4C) and time-course (Figure 4D) of BPAP treatments in 22Rv1 *vs.* LNCaP cells by immunofluorescence. The results indicate that whereas BPAP had an IC_50_ of ~15 μM in 22Rv1, no activity was apparent in LNCaP cells. Time-course analysis showed that, by 8 hrs, AR protein levels are already significantly reduced in 22Rv1 cells, while the 24 hrs time point showed the largest response.

**Figure 5:**
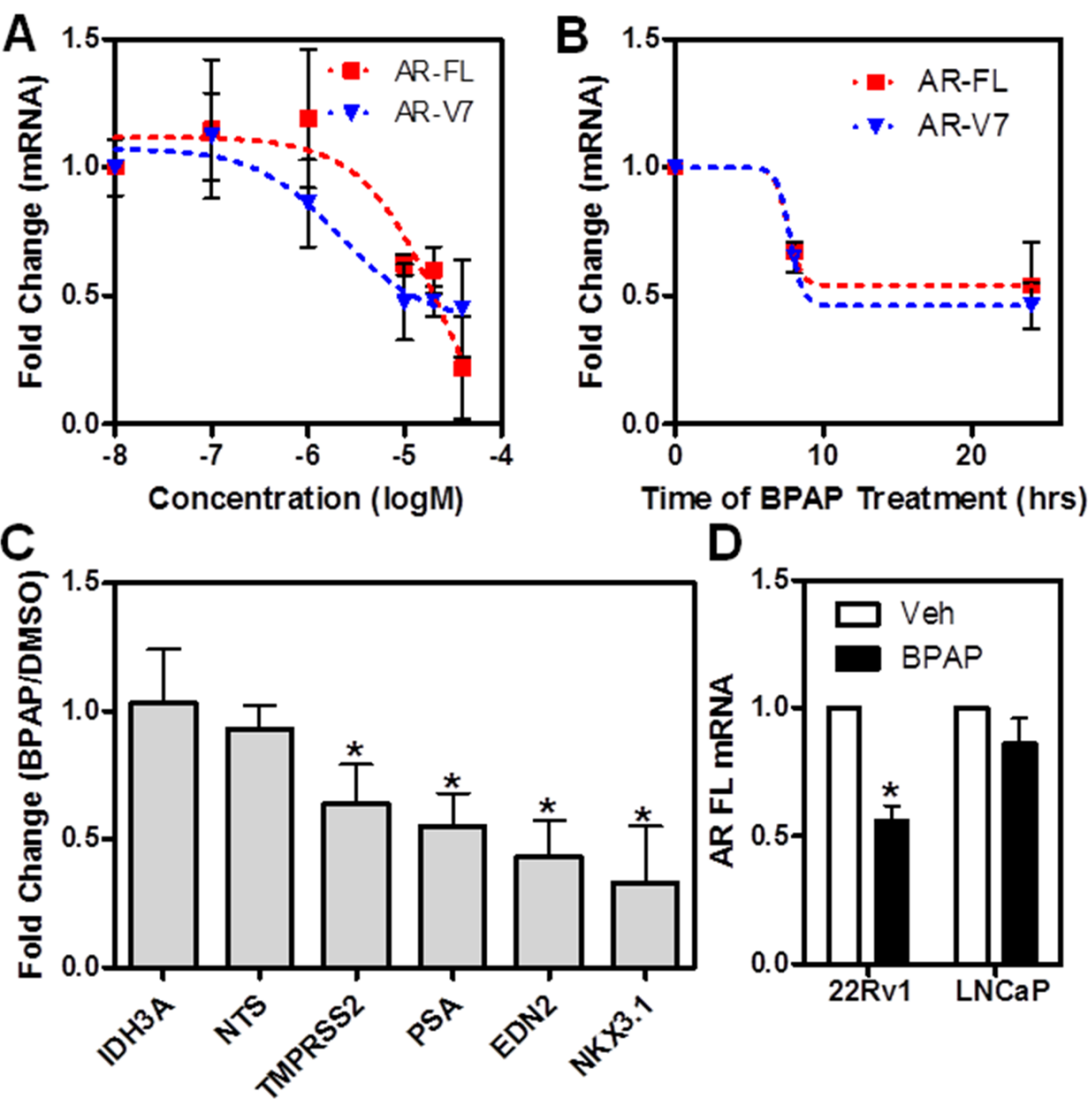
BPAP reduces AR full length and AR-V7 mRNA level in 22Rv1 cells **A–B.** qPCR analysis of full-length AR and AR-V7 mRNA after BPAP dose response (24h) and time course in 22Rv1 cells. **C.** qPCR analysis of AR, AR-V7 target genes and controls after treatment with BPAP for 24 hrs in 22Rv1 cells. **D.** Comparison of the effect of BPAP treatment on AR full length mRNA in 22Rv1 and LNCaP cells as measured by qPCR.

Next, to further validate the effect of BPAP on AR and AR-V7 in CRPC models, we performed immunoblot analysis of whole cell extracts of LNCaP (a “negative control,” as they only express full-length, point mutated AR – indicated by the number 1 in the Western blots in Figure 4E), 22Rv1 (which express a higher molecular weight full-length AR due to duplication of the DNA binding domain – indicated by the number 2 in Figure 4E–4F, and several AR variants, marked by number 3), and the stable GFP-AR-V7:PC-3 (a “positive control” for the AR-V7 antibody, where GFP-AR-V7 has a similar size to the full length AR and is marked by the number 4 in Figure 4E–4F). Again, the effect of BPAP was clear in 22Rv1 where both the full-length and the variant AR were reduced. The fact that no effect was seen in GFP-AR-V7:PC3 suggests that the mechanism of action of BPAP is not simply due to increased protein turnover (see Figure 5 for evidence that the mechanism is transcriptional in 22Rv1).

We also performed immunoblotting to compare the effects of BPAP with BPA treatment in the absence and presence of DHT for 24 hrs (Supplementary Figure S2B). Interestingly, BPAP in combination with DHT lead to partial stabilization of full-length AR, with the levels of AR-variants remaining low, suggesting that BPAP is not a competitive ligand for the AR binding pocket. As expected, the parent molecule BPA had no effect on AR levels in either cell line.

We then asked if the effect of BPAP in 22Rv1 was due to changes in AR mRNA, or if it was a protein destabilization event (or both). As shown in Figure 5, by dose-response (5A), time-course (5B) and in comparison with LNCaP cells (5D), BPAP reduced both AR full-length and AR-V7 mRNA, as measured by qPCR using isoform-specific primers. Collectively, these results are similar to the changes we observed in nuclear protein levels. Extending these results in a functional context, we also examined the effect of BPAP upon the expression of several AR target gene mRNAs, AR-V7 specific targets (*e.g.,* EDN2 [24]) and other non AR related genes as controls (*e.g.,* IDH3A) (Figure 5C). As expected, AR and AR-V7 target gene mRNA was significantly reduced by BPAP treatment.

### BPAP is anti-proliferative and causes reduction in anchorage-independent growth of 22Rv1 cells

**Figure 6:**
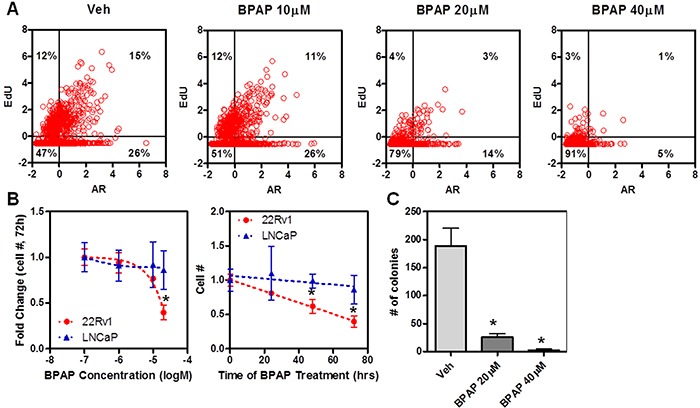
BPAP causes reduction in cell proliferation and cell number in 22Rv1 cells **A.** Single cell analysis of 22Rv1 cells treated with different concentrations of BPAP for 24 hrs and then stained with EdU (y axis) and labeled with AR antibody (x axis). **B.** Cell number analysis after dose-response and time course of treatment with vehicle or BPAP in 22Rv1 vs. LNCaP. **C.** Anchorage independent growth assay was performed in 22Rv1 cells for 21 days of treatment with BPAP and then quantified by counting colonies after crystal violet staining.

**Figure 7:**
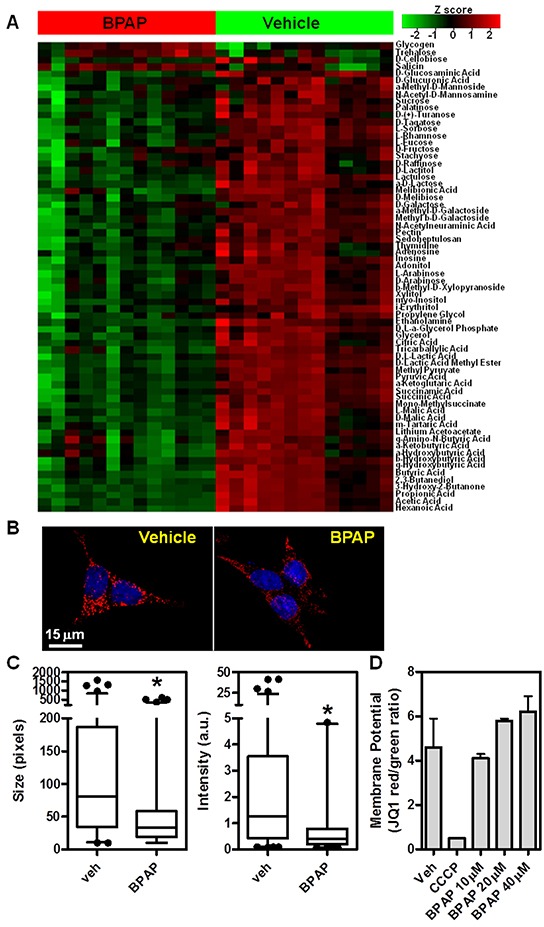
BPAP causes changes in cell metabolism and mitochondria **A.** Metabolomics Phenotype Arrays (sugar sources) were performed in 22Rv1 cells after treatment with vehicle or BPAP 20μM for 24h. Heatmap represents differential carbon source usage between treatments as represented by Z-score, each column represents biological replicates. **B–C.** BPAP reduces mitochondria size and intensity of signal after 24 hr of treatment in 22Rv1 cells. Images were taken with 100x objective on a DeltaVision deconvolution microscope. Size and intensity of mitochondria were measured via automated image analysis routines. **D.** 22Rv1 were treated with BPAP 10–40μM for 24hrs and then stained with JC-1 to measure mitochondrial potential. Graph shows red/green ratio that represents mitochondria functionality. 50μM CCCP was used as positive control.

We next determined the effect of BPAP upon the cell cycle and growth of 22Rv1 cells. We treated cells with different concentrations of BPAP for 24 hrs, pulsed them briefly with EdU to identify which cells are actively engaged in DNA synthesis, and then performed dual labeling with Click-iT EdU kit and anti-AR antibody [3]. Figure 6A shows single cell data in scatter plots; AR levels are represented on the x axis and EdU staining on the y axis. These data further confirm a dose-dependent reduction of AR nuclear levels upon BPAP treatment (x axis), which is accompanied by a reduction in EdU positive cells (y axis), indicating that BPAP induced a marked change in the number of cells entering S phase, from 27% to 4%. If 22Rv1 and LNCaP cells were treated with BPAP with different doses for 24 hrs (Figure 6B), or for longer times (*i.e.*, up to 72 h, Figure 6B), the cell number was reduced by 50-60% only in 22Rv1 cells with a comparable IC_50_ (see Figures 4–5). We also performed a live/dead assay using the Draq5/Draq7 dye combination and the mitochondrial integrity dye JC-1, that measures mitochondrial potential and early signs of apoptosis, to determine if BPAP was causing cell death/apoptosis of 22Rv1 during the 24 hr time period at a dose of 20mM. During this time frame no evident toxicity/apoptosis was observed indicating that BPAP-linked effects during the first 24 hrs are not dependent upon cell death or general toxicity (Figure 7D, and *data not shown*), and that cell cycle arrest may be the main initial mechanism of action. Signs of cell death became more evident after 48 hrs or by increasing BPAP concentration above 20μM.

We then performed a 21-day soft-agar anchorage-independent growth assay and found that treatment with BPAP greatly reduced the number of colonies as determined by crystal violet staining (Figure 6C); of note, the remaining colonies present in the treatment were small, with some evidence of cell debris.

In conclusion, we have identified BPAP as a novel AR down-regulator in CRPC cells that causes cell cycle arrest, and time- and dose-dependent reduction in cell number and soft-agar colony formation.

### BPAP causes reduction in mitochondrial activity

Because of the cell cycle arrest and cell number reduction caused by BPAP, and because it is affecting AR levels, a major player in prostate cancer cell metabolism [25–27], we queried if BPAP was directly affecting mitochondrial activity and cell metabolism. To do so, we took two approaches. First, we applied a metabolomics approach using Metabolomic Phenotypic MicroArrays (Biolog Inc.) to determine if BPAP treatment affected uptake and utilization of ~90 different carbon sources in 22Rv1 cells. In Figure 7A, we treated 22Rv1 cells with 20μM BPAP and after 24 hours we measured carbon source uptake and utilization using this colorimetric platform. The results, represented as a heatmap, clearly show that BPAP caused a reduction in usage of many carbon sources, indicating a substantial alteration in sugar uptake and metabolism. To further explore this, we also labeled mitochondria using the MitoTracker Red dye and found that BPAP caused reduction in labeling intensity and size of mitochondria, as evident from the 100x, z-stack, deconvolved images in Figure 7B and the quantification of single optical planes in Figure 7C. To determine if the reduction in size and intensity of the mitochondria were caused by early signs of apoptosis, we performed live imaging of 22Rv1 cells and stained them with JC-1, a dye that measures mitochondrial potential; reduction of JC-1 labeling is symptomatic of apoptosis. As shown in Figure 7D, at 24 hrs, BPAP did not cause reduction of mitochondrial potential, thus ruling out pro-apoptotic effects. The oxidative phosphorylation inhibitor CCCP was used as a positive control for the assay.

In conclusion, utilizing HTM/HCA, we have identified the Bisphenol A analog, BPAP, as a novel molecule capable of reducing the levels of AR (both full-length and variants) in CRPC tumor cell models *in vitro*. BPAP causes cell cycle arrest and metabolic changes in keeping with its action as a down-regulator of AR. This endocrine disruptor thus represents an interesting new compound with potential for structure/activity studies to develop new lead candidate moieties for CRPC.

## DISCUSSION

The Androgen Receptor (AR) has been the top drug target in prostate cancer for decades, as it plays central roles in the development and progression of such tumors [1, 5, 28]. The vast majority of therapeutic strategies for prostate cancer patients are designed to block AR activity, by either reducing systemic and/or local production of androgens (GnRH agonists, 17α-hydroxylase inhibitors) or by direct competition for the ligand binding pocket of AR (AR antagonists). These therapies initially work but lose efficacy when the tumor evolves to its castrate resistant form (CRPC); unfortunately, CPRC remains incurable and lethal, despite some recent success of next generation anti-androgens Abiraterone and Enzalutamide. Importantly, these advanced tumors remain dependent upon AR [5, 28–32]. Many mechanisms have been proposed on how these tumors become drug resistant while keeping AR dependency, including: 1) persistent local androgen production, 2) AR over-expression, 3) signaling pathways, and 4) AR ligand binding domain point mutations [28–33]. However, one of the most intriguing ways for AR dependency of the tumor even in complete absence of ligand is the increased expression of many AR splice variants (AR-Vs) which can be constitutively nuclear and transcriptionally active, and lack the LBD [6–8]. To counteract AR-V actions, new therapeutic strategies are needed as their expression correlates with a poor prognosis [7, 9, 34]. One of the potential strategies to reverse AR activity in CRPC would be to reduce the expression of AR; indeed, knock-down of AR-V7 in 22Rv1 cells reduced growth of mouse xenografts [35].

In this study, we were interested in utilizing a single cell–based high throughput microscopy platform to characterize modulators of AR levels and localization by comparing the effects of small molecules on endogenous AR across six prostate and breast cancer cell lines. Importantly, this effort was designed to study the effects of compounds on *endogenous* AR, without the need for engineered cell systems. After demonstrating the capability of this multi-cell line platform for classifying known AR antagonists and determining their cell specific activities, we tested a small number of endocrine disrupting compounds, thus beginning to exploit the highly varied chemical space derived from the chemical industry, a largely untapped resource for drug discovery. AR is a well known endocrine disruptor target (*e.g.,* BPA, Vinclozoline); furthermore, a BADGE (Bisphenol A Diglycidic Ether) marine sponge metabolite (EPI-001) has been shown to be effective in reducing CRPC growth via non-canonical mechanisms [15].

In our primary screen, we identified several EDCs with androgenic or anti-androgenic activity (at least in terms of AR nuclear levels and stabilization), however, one BPA analog, Bisphenol AP (BPAP), stood out in our analysis as it caused marked reduction of AR levels specifically in 22Rv1 cells. BPAP is commonly used as a plasticizer and a component of flame retardants in many industrial products, and is a bona fide environmental contaminant, especially in wastewater [36].

We validated BPAP effects in several CRPC models and found that, indeed, it reduces both AR full-length and AR splice variants both at the protein and mRNA level. Interestingly, DHT treatment was still capable of stabilizing full length AR thus suggesting that BPAP does not act as a competitive ligand. Moreover, when we used PC-3 cells that were engineered to express GFP-AR-V7, we did not see any effect on AR protein levels after BPAP treatment, also suggesting a lack of direct interaction with AR (N-terminus in this case) or a protein degradation mechanism (and highlighting that BPAP blocks AR gene transcription). While BPAP is not a very potent compound (IC_50_~20μM) and would require SAR experiments to optimize its potency and possibly selectivity (since it shows some toxicity at concentrations above 40μM and can bind other nuclear receptors – *i.e.,* estrogen receptors, [14]), it was still quite efficient in blocking the cell cycle. Interestingly, BPAP also affected mitochondria and cell metabolism, suggesting it is a compound with multiple mechanisms of action. Although it is likely that many BPAP targets exist in the cell, they remain poorly characterized. It is reasonably clear, however, that BPAP shows some specificity for CRPC cell lines, thus identifying BPAP as a potentially interesting lead compound for this disease, and also emphasizes that the large chemical space covered by industry-linked chemical syntheses may have additional biomedical utility.

In conclusion, we developed a high throughput, microscopy based platform to classify the effect of compounds on endogenous AR and we identified BPAP, a bisphenol A analog, as a molecule that is capable of causing AR (both full-length and variants) down-regulation in CRPCs resulting in cell cycle arrest and metabolic defects. Further studies will be needed to fully unravel BPAP mechanism(s) of action and therapeutic potential via SAR studies.

## MATERIALS AND METHODS

### Cell culture, compound treatments, qPCR, Western blot, and anchorage-independent growth assays

Cell lines (MCF-7, LLC2, T47D, LNCaP, 22RV1, LNCaP95, VCaP, and LNCaP/C4-2) were cultured as described by ATCC. Cells were plated either on optical quality 384-well glass bottom plates (Greiner Bio-One, Monroe, NC) or plastic bottom plates (Brooks Automation, Fremont, CA) using a TitreTek robot (Thermo Fischer Scientific, Waltham, MA). LNCaP95 were a kind gift from Dr. Stephen R. Plymate (University of Washington) and GFP-AR-V7:PC-3 were obtained from Dr. Marco Marcelli (Baylor College of Medicine), single cell cloned and used as in [37]. After 24 hrs, cells were treated with compounds at the concentrations indicated, in quadruplicate wells per condition; every plate contained several wells treated with vehicle (DMSO) or DHT 1nM, used as plate controls. The compounds were obtained from multiple manufacturers (information available upon request). RNA extraction and qPCR were performed as in [11], primer sequences are in Supplementary Table S1. Western blots and anchorage-independent growth assays were performed following standard protocols.

### Immunolabeling, EdU and mitochondria staining

Cells were fixed in 4% paraformaldehyde for 30 minutes on ice and quenched with 0.1 M ammonium chloride. Permeabilization was performed using 0.5% Triton-X100 for 30 minutes. Cells were blocked with 5% milk in TBS-T buffer and then incubated at 4°C overnight with the following antibodies: AR (AR441, gift from Drs Weigel and Edwards, BCM; AR N-20, Santa Cruz Biotechnologies, Dallas, TX) and AR-V7, (Bethyl Laboratories, Montgomery, TX; Abcam, San Francisco, CA). Following 3x washes, cells were incubated for 30 minutes with Alexa Fluor dye-tagged secondary antibodies (Thermo Fisher, Waltham, MA) at room temperature. Cells were washed and a post-fixation step was performed. Finally, cells were incubated for 5 minutes with 1 μg/ml DAPI. All immunolabeling in multi-well plates was carried out robotically using a BioMek NX instrument (Beckman Coulter, Indianapolis, IN).

EdU staining, after a 5 minute pulse, was performed following manufacturer's instructions (Thermo Fisher, Waltham, MA). For mitochondria staining we used Mitotracker Red CMX (Thermo Fisher, Waltham, MA), cells were exposed to the dye for 30 minutes. JC-1 (Thermo Fisher, Waltham, MA) treatment was performed in live cells following manufacturer's instructions.

### Automated microscopy, image processing and analysis

384 well plates were imaged on an IC200 (Vala Sciences, San Diego, CA) or InCell 6000 (GE Healthcare, Piscataway, NJ) high throughput microscope, using 40X/0.95 NA lenses. Images were background subtracted using a rolling-ball method and segmented using the DAPI channel applying a locally adaptive mean threshold. Objects that had an area of <20 pixels were considered noise and discarded. A distance transform was applied to the nuclear objects and used in a seeded watershed routine to split touching nuclei and produce the final nuclear masks. Approximate cell masks were obtained by dilating a circular region around the nuclei. Regions with cell masks touching the edge of the image were discarded. Finally, a set of morphological and intensity based measurements [3, 13, 18] were extracted from antibody and DNA-labeled channels. These steps were performed using PipelinePilot 8.5 (Accelrys Inc., San Diego, CA) with the Imaging and Statistics Toolboxes. Additional imaging was performed with a Deltavision image restoration system (GE Healthcare, Piscataway, NJ).

### Metabolomic phenotypic microarrays

The metabolic phenotyping was performed as previously reported [26]. Briefly, 22Rv1 cells were treated for 24 hrs with vehicle or 20 μM BPAP and then seeded overnight in nutrient containing plates (Biolog Inc., Hayward, CA). Metabolite utilization was measured in real time by the extent of reduction of tetrazolium dye via spectrophotometry. Data points were corrected by subtracting average value of the negative controls (empty wells) and then comparisons were made via heat maps.

## SUPPLEMENTARY FIGURES AND TABLE


