# Supplementary material for: High throughput microscopy identifies bisphenol AP, a bisphenol A analog, as a novel AR down-regulator
Source: Oncotarget. 2016 Feb 24;7(13):16962–74. doi: 10.18632/oncotarget.7655 (PMC4941363; doi:10.18632/oncotarget.7655)
Supplement: Supplementary file 1 [file oncotarget-07-16962-s001.pdf]

SUPPLEMENTARY FIGURES AND TABLE

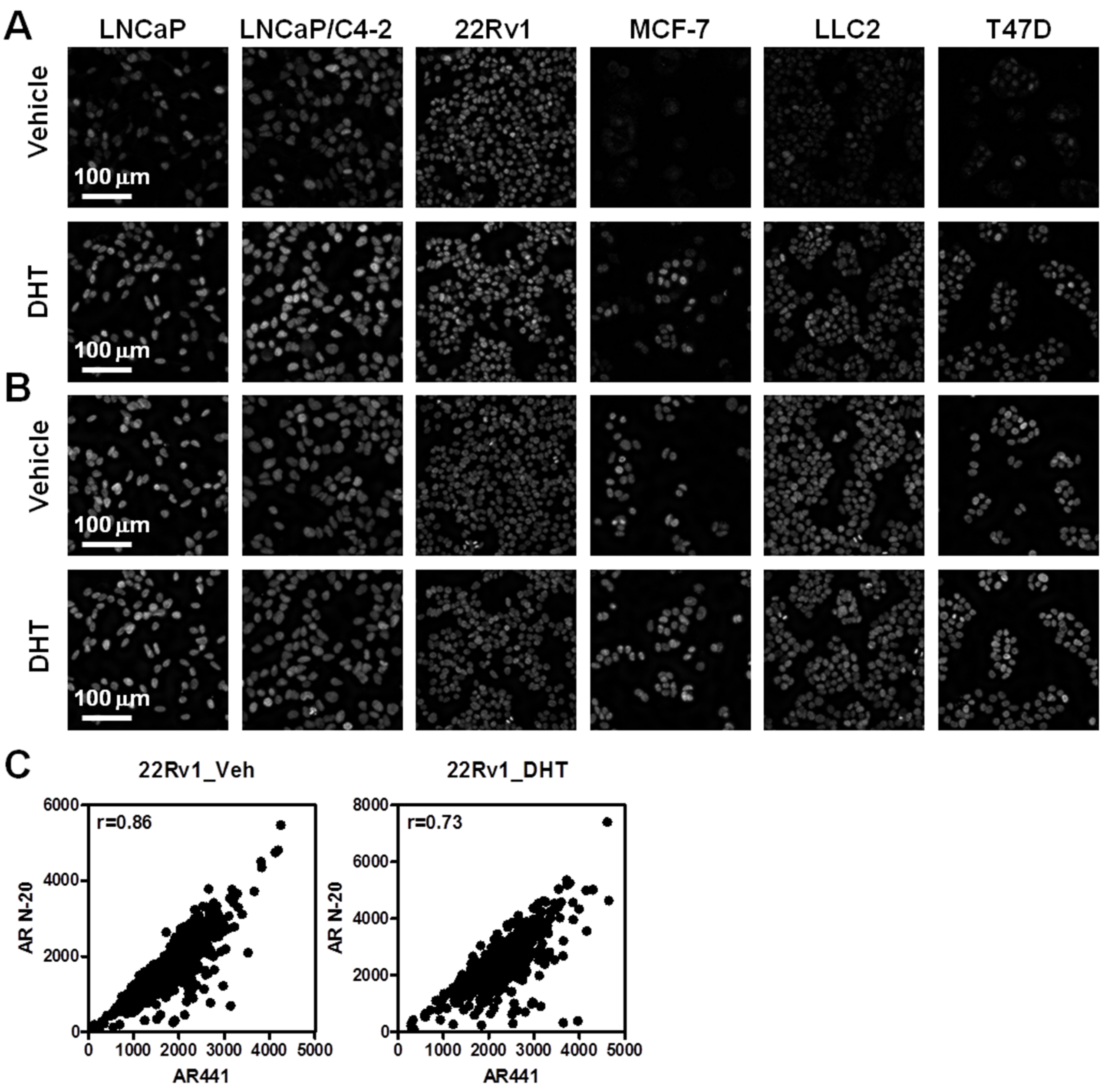

Supplementary Figure S1: High throughput microscopy-based analysis of endogenous AR nuclear level and translocation across prostate and breast cancer cell lines. **A–B.** Representative images of the cell lines used in HTM labeled with AR N-20 antibody or stained with DAPI after 24 hrs of treatment with either vehicle or 1 nM DHT. **C.** Spearman correlation analysis between AR441 and AR N-20 after dual immunofluorescence in 22Rv1.

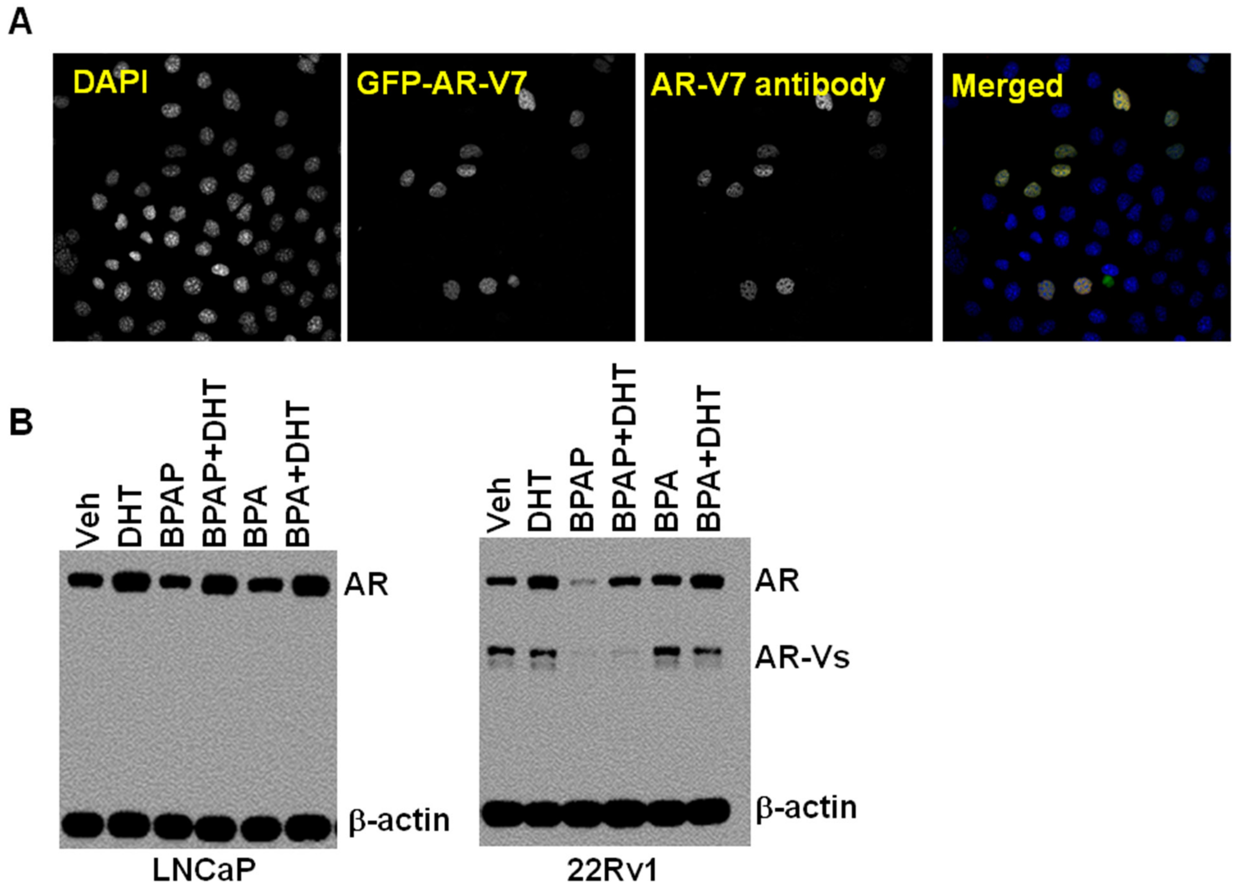

Supplementary Figure S2: Validation of AR-V7 antibody by imaging and effect of BPA on AR-V7. A. GFP-AR-V7:PC-3 were treated with vehicle or 20 µM BPAP for 24 hrs and immunolabeled with AR-V7 antibody. **B.** Western blot in LNCaP and 22Rv1 showing reduction of AR (full length and variants) only in 22Rv1. Cells were treated with 20 µM BPA or BPAP for 24 hrs +/− DHT.

Supplementary Table S1: qPCR primer sequences

Gene	Forward	Reverse
AR	cggaagctgaagaaacttgg	atggcttccaggacattcag
AR-V7	caccatggaagtgcagttagggctgggaagggtctaccct	tcagggtctggtcattttgagatgcttgcaattgcc
EDN2	tgggtgaacactcctgaaca	aggcagaaggtggcacag
IDH3A	agccggtcacccatctatgaa	cttctgtgttctctcgaatggtc
NKX3.1	gagacgctggcagagacc	tccaacagataagaccccaag
NTS	gcatgctactcctggctttc	ccaagagggaacatgtgctt
PSA	tccctatgggatcagactgc	tgggtaggtggagaatggag
TMPRSS2	ggtagtactgagccggatgc	caccaccagctattggacct
